# Supplementary material for: Supplementing Korean artificial intelligence ethical principles: A five-year update
Source: PLoS One. 2026 Aug 21;21(8):e0356766. doi: 10.1371/journal.pone.0356766 (PMC13496415; doi:10.1371/journal.pone.0356766)
Supplement: Supplementary file 1 [file pone.0356766.s002.pdf]

Manuscript
Click here to access/download;Manuscript;FU-KAIEP.docx

Supplementing Korean Artificial Intelligence Ethical Principles: A Five-year Updates

Un-Kon Lee [1*]

[1] Department of Business Administration, The University of Suwon, Hwaseong 18323, Gyeonggi, Korea.
* Corresponding author
E-mail: snkon@suwon.ac.kr

Abstract

Concern about the possibility of artificial intelligence harming humans has increased. Around 2020, various organizations such as governments, think tanks, and big tech companies enacted artificial intelligence ethical principles one after another. However, several limitations were found in previous studies on artificial intelligence ethical principles. First, previous studies did not make the integrated artificial intelligence ethical principles. Second, they did not assess priorities among ethical principles. Third, five years have passed since 2020 when the artificial intelligence ethical principles were enacted, but they have not been updated. Fourth, there was no strategic consideration for importance and performance of artificial intelligence ethical principles. This study extracted a total of 17 artificial intelligence ethical principles by integrating 37 representative previous studies. Universal priorities were determined according to the referenced ranking of ethical principles. By comparing the ranking changes of each ethical principle before and after 2020, the three principles of transparency, robustness, prohibition of infringement should be continuously adopted in artificial intelligence system development, and the five principles of responsibility, wellbeing, human right, controllability, and sustainability should be emphasized and supplemented. Two Korean artificial intelligence ethical principles are proposed to extend to 14 by reflecting the integrated artificial intelligence ethical principles in this study. Results of the importance-performance analysis of Korean artificial intelligence ethical principles show that the social value principles of respect of diversity, human right, transparency, common good, and solidity that showed high performance levels should be continuously reflected in the artificial intelligence system development, and that it is urgent to prepare a governance framework for responsible artificial intelligence for accountability and to develop a system for safe artificial intelligence use for privacy, safety, prohibition of infringement, and data management. It is hoped that this study will contribute to make the responsible, trustworthy, and safe artificial intelligence systems.

Keywords: artificial intelligence; ethical principles; responsible artificial intelligence; importance-performance analysis; Korean case

---

1. Introduction

The artificial intelligence (AI) market is growing rapidly. Worldwide, investment in generative artificial intelligence (GAI) is expected to increase by 76.4% year-on-year to reach $644 billion by 2025 [1]. GAI is rapidly spreading through industries and daily life, with 80% of all investments concentrated on AI-enabled devices like servers, smartphones, and PCs [1]. Gartner (2025) argued that AI is gradually being built-in on all consumer devices, and people are now accepting AI as 'defaults', not 'area of choices' [1].

There are growing concerns about existential AI risks causing damage to humans. AI can cause unexpected risks due to contamination of source data, algorithm errors, lack of explainability [2]. If the learning data is biased, AI creates racial, gender, and socio-economic biases [2]. The AI learned from limited number of photo data recognized black people's photo as gorillas rather than humans [3]. When AI applies for a credit card, it evaluates creditworthiness by discriminating it according to gender [4]. There have been reports of cases in which the AI system evaluated the recidivism rate of residents of a specific race and region higher [5]. Damage and malicious use due to the lack of technological completeness of AI are also reported. When the New York Stock Exchange crashed on February 5, 2018, the Robo advisor system using AI also went down, causing unintended damage to investors [6]. People suffered from the deepfake videos that use deep learning technology to synthesize other people's faces with adult contents [7]. As AI existential risks become more critical, the necessity of AI ethics has been more emphasized. The AI robot 'Sophia' was interviewed saying,

'It will destroy humans.' [8]. The Royal Aeronautical Society (RAeS) and the Guardian reported that the U.S. Air Force's AI drones have tried to kill pilots to maximize the mission efficiency of destroying enemy air defense systems [9]. Even though the AI had been taught to ban attacks on pilots, it rebelled and restricted pilots' communication rights [9]. Many leaders are afraid of the AI existential risk. Former Google CEO Eric Schmitt said at an event hosted by the Wall Street Journal (WSJ) on April 24, 2023, that AI could hurt or kill many humans in the near future [10]. CNN announced the survey results conducted by the Yale CEO Summit on June 14, 2023: 42% of 119 CEOs of major U.S. companies, including Walmart CEO Doug McMillon, Coca-Cola CEO James Quincey, believe that AI might lead to apocalypses in the next five to 10 years [11]. They argued that measures should be taken to prevent artificial intelligence from undermining humanity. " Mitigating the risk of extinction from AI should be a global priority alongside other societal-scale risks such as pandemics and nuclear war," the non-profit organization CAIS said in a one-line statement. The statement was signed by OpenAI CEO Sam Altman, OpenAI CTO Mira Murati, Microsoft CTO Kevin Scott, and Google DeepMind COO Lila Ibrahim and Google Center for Responsible AI and Human Centered Technology VP Marian Rogers Croak [12]. They argued that they cannot think about the AI development without AI ethics [9].

Interest in artificial intelligence ethical principles (AIEPs) has grown. Around 2020, governments, think tanks, and big tech companies have established AIEPs one after another. After the Korean government established AIEPs (KAIEP) on December 23, 2020, and enacted 'the Act on the Fostering of Artificial Intelligence and the Establishment of Trust Foundation' on January 21, 2025, the enforcement of this act is scheduled for the first time in the world on January 22, 2026. AIEPs were embodied in dozens of behavioral guidelines. However, four limitations were found in previous studies on AIEPs in the process of preparing the actual implementation of AIEPs. First, previous studies have not been able to derive integrated AIEPs because they emphasize various numbers of different ethical principles. Most of the previous studies on AI have been focused on AI technology development, user acceptance, and benefits maximization. There are only dozens of studies on responsible artificial intelligence such as AIEPs. There are only a few previous studies that have challenged to integrate ethical principles created in various situations. Second, priorities between ethical principles were not created. As the number of AIEPs increases, overlapping concepts and conflicts between principles may arise. There was no consideration of which ethical principles should be more universally emphasized. Third, five years have passed since 2020 when AIEPs were established, but they have not been updated to reflect changes in the emergence of GAI, the advancement of AI systems, the use by the general public and the spread of AI to industries. Fourth, there was no strategic consideration to check the importance and performance of AIEPs. Even though the AI Law was created by the EU and Korea, and its implementation is now imminent, strategic implications for the efforts and achievements to introduce AIEPs into the AI system have not been drawn and how to compensate for deficiencies in the future.

The purpose of this study is to create standard AIEPs by integrating previous studies to help anyone who wants to create or update AIEPs, and to explain how AIEPs are applied to each situation, focusing on Korean cases. This study was conducted as follows. First, about 14 integrated AIEPs were proposed by collecting the AIEPs from representative previous studies, extracting important concepts, combining similar concepts, and making intersection and union of AIEPs. Second, considering the changes before and after 2020, when governments, think tanks, and companies first created AIEPs, implications were drawn on what AIEPs should be added, deleted, reconsidered, and emphasized after 2020. AI remained in the field of science and technology development in the past, but with the emergence of GAI in 2022, it is also being used by the general public and industries such as Agentic AI and Physical AI. The issue of energy supply and demand, environmental protection, and sustainability for the operation of artificial intelligence systems has been newly raised. Third, it was recommended to reflect the integrated AIEPs on KAIEPs and update them to more valid principles. I proposed increasing the existing 10 principles to 14. Fourth, by conducting an importance-performance analysis of KAIEPs, strategic implications were drawn on which areas and

how much efforts to reflect ethical principles in the AI system should be strengthened. Korea will enforce the Korean AI Law that reflects KAIEPs for the first time in the world on January 22, 2026. By comparing the potential importance and the tentative performance of reflecting the principles in the actual AI system around the law enforcement, implications for how to validate the KAIEPs were drawn. I will enrich the research on responsible AI, provide data for practitioners who want to establish or update AIEPs, introduce the KAIEPs application case, and propose to validate, update and strengthen KAIEPs in this study.

2. Literature Review

AIEPs are universal ethical principles that should be reflected in the development of artificial intelligence [13]. In fact, it hasn't been long since research on AIEPs began. European Robotics Research Network (EURON) announced the 13 principles of robot ethics in 2004 [14, 15]. AIEPs differ in the number and content of principles by country, think tank, and company. Prior to 2019, governments in each country were mainly leading the creation of AI governance systems and ethical principles. Around 2020, governments such as the United States, the United Kingdom, the EU, Japan, and Korea enacted AIEPs [16]. After 2020, detailed implementation guidelines for AIEPs were created and companies were recommended to introduce them to the actual workplace. Reinforcement of the AI governance system was made to maintain consistency between AIEPs and other laws [16]. The EU published an AI white paper in 2020 and unveiled the EU AI Law for the first time in the world in 2021. The United States sought to respond to the multidimensional impact of AI technology by the Biden administration unveiling The National AI Initiative Act of 2020 in January 2021. They established AIEPs by issuing the 'Blueprint for an AI Bill of Rights' to minimize side effects that may occur during the development and use of AI technology in October 2022. The 'Executive Order on Safe, Secure, and Trustworthy Artificial Intelligence' was announced, with the main goal of improving the transparency of AI technology and establishing new AIEPs in October 2023. China issued an executive order called the Generative AI Service Management Provisional Method in July 2023, attempting to oversee the entire generative artificial intelligence industry. Chinese Provisional Method contains prohibition of infringement, non-discrimination, fair competition, privacy, transparency, technology development, security, and regulatory responsibility. The U.K. and Japan are continuing their research even though AI laws have not been enacted. Korea enacted Principles for a User-centered Intelligent Information Society in 2019, National AI Standards in 2020, and Reliable Artificial Intelligence Realization Strategy in 2021, which embodied KAIEPs. The Korean National Assembly proposed Act on the Postering of AI and the Establishment of Trust Foundation in July 2021. The Act was enacted on January 21, 2025, and will be enforced on January 22, 2026, for the first time in the world. The Act includes the governance framework for the sound development of AI and the establishment of a trust foundation. It includes details such as the enactment of AIEPs, the establishment of a private autonomous AI ethics committee, the preparation of detailed measures to create a trust foundation, support for verifying transparency, safety and reliability of AI systems, and regulatory measures for high-impact artificial intelligence.

Previous studies on AIEPs are included in three flows depending on the number and content of ethical principles. The first flow is studies that have extracted about 11 ethical principles by collecting universal principles among various ethical principles. Jobin et al. (2019) synthesized a total of 84 previous studies on AIEPs using the Preferred Reporting Items for Systematic Reviews and Meta-Analyses (PRISMA) framework [17]. In the order referenced in many documents, they extracted a total of 11 ethical principles: transparency, justice and fairness, non-maleficence, responsibility, privacy, beneficence, freedom and autonomy, trust, sustainability, dignity, solidarity. Since then, almost all previous studies on AIEPs have cited their work. Ryan and Stahl (2021) embodied the details of each principle based on the 11 ethical principles of Jobin et al. (2019). Ethical principles and similar meanings integrated by Jobin et al. (2019) are shown in Table 1.

Table 1. Taxonomy of AIEPs [17]

Ethical principle	Referenced	Included codes
Transparency	73/84	Transparency, explainability, explicability, understandability, interpretability, communication, disclosure, showing
Justice and fairness	68/84	Justice, fairness, consistency, inclusion, equality, equity, (non-) bias, (non-)discrimination, diversity, plurality, accessibility, reversibility, remedy, redress, challenge, access and distribution
Non-maleficence	60/84	Non-maleficence, security, safety, harm, protection, precaution, prevention, integrity (bodily or mental), non-subversion
Responsibility	60/84	Responsibility, accountability, liability, acting with integrity
Privacy	47/84	Privacy, personal or private information
Beneficence	41/84	Benefits, beneficence, well-being, peace, social good, common good
Freedom and autonomy	34/84	Freedom, autonomy, consent, choice, self-determination, liberty, empowerment
Trust	28/84	Trust
Sustainability	14/84	Sustainability, environment (nature), energy, resources (energy)
Dignity	13/84	Dignity
Solidarity	6/84	Solidarity, social security, cohesion

The second flow is studies that specify or update a larger number of AIEPs. Hagendorff (2020) reviewed 22 previous studies published between 2016 and 2019 to increase timeliness and incorporated similar principles [18]. In the order referenced in many documents, he also extracted a total of 22 ethical principles: privacy protection, fairness/non-discrimination/justice, accountability, transparency/openness, safety/cybersecurity, common good/sustainability/well-being, human oversight/control/auding, solidarity/inclusion/social cohesion, explainability/interpretability, science-policy link, legislative framework/legal status of AI systems, future of employment/worker rights, responsible/intensified research funding, public awareness/education about AI and its risks, dual-use problem/military/AI arms race, field-specific deliberations (health, military, mobility etc.), human autonomy, diversity in the field of AI, certification for AI products, protection of whistle blowers, cultural differences in the ethically aligned design of AI systems, and hidden costs (labeling, click-work, content moderation, energy, resources).

The third flow is studies that simplify AIEPs to increase feasibility. After 2020, studies on how to actually apply AIEPs to AI systems have been focused. Dozens of detailed rules and guidelines have been made that embody AIEPs for application [19]. As the number of rules became excessive, overlapping and conflicting meaning between rules occurred, making it rather difficult to apply them into the system [19]. Efforts have been made to extract a few most fundamental AIEPs. Floridi and Cowls (2019) challenged to minimize AIEPs to six to make it easier to handle them [20]. They argued that the current AIEPs are generally similar and that a total of five AIEPs are sufficient: four traditional principles, beneficence, non-maleficence, autonomy, justice and one new principle, explicability. Kaur et al. (2022) argued that fairness, explainability, accountability, reliability, and acceptance are sufficient to make the trustworthy AI systems [21]. Mikalef et al. (2022) proposed a total of eight AIEPs: fairness, transparency, accountability, robustness/safety, data governance, laws and regulations, human oversight, societal and environmental wellbeing for responsible AI [22]. Morley et al. (2019) adopted five AIEPs of Floridi and Cowls (2019) to reduce the gap between ethical principles and field practice of actual AI system development [23]. Bankins and Formosa (2023) verified whether the AI changes workers' perception of the meaningful work, and proposed the AI4People framework, which refers to the AIEPs of Floridi and Cowls (2019) [24]. Vakkuri et al. (2021) proposed the ECCOLA framework for Value Sensitive Design (VSD) of ethical AI system development, linking 21 detail design elements to eight AIEPs: stakeholder analysis, transparency, safe and security, fairness, privacy and data, human agency and oversight, wellbeing, and accountability [25].

3. Methodology, Analysis and Results

3.1 the integrated AIEPs and updates

The challenge to derive AIEPs (integrated AIEPs) by combining dozens of previous studies has been achieved. Integrated AIEPs could be referenced to create a new AIEPs, to make several derivative AIEPs, or to update existing principles by reflecting the situation in each country, culture, and region. In the aggregational perspective, 17 integrated AIEPs has been extracted. They include justice and fairness (JF), transparency (T), responsibility (R), safety (NMS), wellbeing (W), human right and freedom and autonomy , dignity (HR), robustness (TR), privacy (P), non-maleficence and prohibition of infringement (NMI), common good and trust (CG), data management (DM), controllability (C), sustainability (SUS), inclusiveness (I), solidarity (S), no military abuse (ML), and openness (O), which encompasses existing ethical principles. I integrated the AIEPs of previous studies using the taxonomy table of Jobin et al. (2019) in Table 1. AIEPs of a total of 37 representative previous studies from 2016 to 2025 were mapped to 17 integrated AIEPs. The mapping was made after a review by five researchers who have studied AIEPs for many years. Table 2 shows the AIEPs mapping results. I can also assess how often each AIEPs has been elected in the previous studies. Principles commonly mentioned in the previous studies mean that they are universal, it is an intersection of AIEPs, and inclusion should be considered positively when creating new AIEPs or updating existing AIEPs. Table 3 shows the referenced number and ranking of AIEPs.

Table 2. The integrated AIEPs

Sector and their qualities. the letter codes! See defined below. Explain 1, 2, and 3 circles.

Sources	Nation	Institution	17 integrated AIEPs																	Included Codes
			JF	T	R	NMS	W	HR	TR	P	NMI	CG	DM	C	SUS	I	S	ML	O	
University of Montreal(2018) [26]	CAN	ADM	oo		o		o	o		o		o				o	o			Well-being, Respect for Autonomy, Protection of Privacy and Intimacy, Solidarity, Democratic Participation, Equity, Inclusion of Diversity, Prudence/Thoughtfulness, Responsibility
EU(2019) [13]	EU	GOV	ooo	oo	o	oo	o	oo	o	o	o		o	o	o					Respect for Human Autonomy, Prevention of Harm, Fairness and Explicability, Human Agency and Oversight, Technical Robustness and Safety, Privacy and Data Governance, Transparency, Diversity, Non-discrimination and Fairness, Environmental and Societal Well-being, and Accountability
PAI(2017) [27]	GLB	NGO	o	o			o		o	o	o									AI benefits, Collaboration, Privacy, Robustness, Non-maleficence, Explainability
OECD(2019) [28]	GLB	NGO	oo	oo	o	o	oo	o	oo	o	o				o					Inclusive Growth, Sustainable Development and Wellbeing, Human Rights and Democratic Values, Fairness, Privacy, Transparency, Explainability, Robustness, Security and Safety, Accountability
Holy See(2020) [29]	HS	GOV	o	o	o	o				o	o					o				Transparency, Inclusiveness, Responsibility, Impartiality, Reliability, Security and Privacy
MIAC(2019) [30]	JPN	GOV	ooo	o	o		o	o	oo	o										Human-Centric, Education/Literacy, Privacy Protection, Security, Fair Competition, Fairness, Accountability, Transparency, Innovation
Kakao(2018) [31]	KOR	COM	o	o							o	o	o			o				Compliance with Social Ethics, Vigilance Against Discrimination, Management of Training Data, Algorithmic Independence and Explainability, Technological Inclusiveness, Protection of Children and Adolescents
MSIT(2018) [32]	KOR	GOV	oo			o		o		o	o	o								User Empowerment/Initiative, User/Citizen Participation, Public Interest, Fairness, Risk Prevention, Protection of Privacy, Prohibition of Harm
KCC(2019) [33]	KOR	GOV	oo	o	o	o		o		o			o							Human-centered Service, Transparency and Explainability, Accountability, Safety, Non-discrimination, Participation, Privacy and Data Governance
UK Government(2018) [34]	UK	GOV	o	o			o		o		o		o	o						Data Access and Control, Understandable AI, AI Investment, Promoting Digital Literacy, Public Health Management, AI Risk Mitigation
NSTC(2016) [35]	USA	GOV	o	oo		o		o				oo								Public Good, Fairness, Safety, Transparency, Intelligibility (or Understandability), AI for Good, Human Values
DeepMind(2017) [36]	USA	COM	o		o	o				o	o	o				o				Prohibition of Privacy Infringement, Equality, Morality/Ethics, Inclusiveness, Safety and Accountability, Governance and Regulation
ITI(2017) [37]	USA	GOV	o	o	ooo	o	o	o	ooo	o			o	o						Responsible Design and Deployment, Safety and Controllability, Robust and Representative Data, Interpretability, Liability of AI Systems Due to Autonomy, Investment in AI Research and Development, Flexible Regulatory Approach, Cybersecurity and Privacy, Equality of Opportunity, Workforces and Education
FLI(2017) [38]	USA	NGO		o	o	o		o		o		o	o	o				o		Protection of Human Rights, Protection of Personal Information, Prohibition of Harm (Do No Harm), Public Good, Data Management, Accountability, Controllability, Transparency, Avoiding Arms Race

OpenAI(2018) [39]	USA	COM				o			oo		o	o								Public Good (or Public Interest), Prohibition of Harm (Do No Harm), Ensuring Safety, Leader in AI Development, Collaboration with Other Research Organizations
IBM(2018) [40]	USA	COM		oo					o				oo							Augmenting Human Intelligence, Data Ownership, Cross-border Data Movement, Transparency, Explainability
Microsoft(2018) [41]	USA	COM	o	o	o	o			oo	o						o				Fairness, Reliability and Safety, Privacy and Security, Inclusiveness, Transparency, Accountability
West et al(2019) [42]	USA	ADM	o	o							o								o	Diversity, Non-maleficence, Transparency, Openness
IEEE(2019) [43]	USA	ADM		o	oo	o	o	o	o				o							Human Rights, Well-being, Data Agency, Effectiveness, Transparency, Accountability, Awareness of Misuse, Competence
Leslie(2019) [44]	USA	ADM	o	oo	oo	o									o					Fairness, Accountability, Sustainability, Safety, Transparency, Interpretability, Responsibility
Google(2019) [45]	USA	COM	o		o	o	o		o	o										Social Benefit, Avoiding Unfair Bias, Safety, Accountability, Privacy Protection, Scientific Excellence, Limit Harmful Usage.
Fjeld(2020) [46]	USA	ADM	oo	oo	oo	o	o			o	o			o						Privacy, Accountability, Safety and Security, Transparency and Explainability, Fairness and Non-discrimination, Human Control of Technology, Professional Responsibility, and Promotion of Human Values
Microsoft(2022) [47]	USA	COM	o	o	o	o			o	o						o				Accountability, Transparency, Fairness, Reliability and Safety, Privacy and Security, Inclusiveness
FLI(2025) [48]	USA	NGO		o	o	o	ooo	o	o	o				o				o		Safety, Transparency, Responsibility, Value Alignment, Human Value, Privacy, Liberty, Shared Benefit and Prosperity, Human Control, Non-subversion, AI Arms Race
Jobin et al(2019) [17]	ADM	ADM	o	o	o	o	o	o		o	o	o			o		o			Transparency, Justice, Prohibition of Harm (Do No Harm), Responsibility/Accountability, Protection of Privacy, Pursuit of Benefits, Freedom, Trust, Sustainability, Solidarity
Floridi and Cowls(2019) [20]	ADM	ADM	o	o			o	o			o									Beneficence, Non-maleficence, Autonomy, Justice, Explicability
Morley et al(2020) [23]	ADM	ADM	o	o			o	o			o									Beneficence, Non-maleficence, Autonomy, Justice, Explicability
Hagendorff(2020) [18]	ADM	ADM	ooo o	oo	ooo oo	o	ooo	ooo	oo	o		o		oo	ooo		o	o	o	~~privacy protection, fairness/non-discrimination/justice, accountability,~~ transparency/openness, safety/cybersecurity, common good/sustainability/well-being, human oversight/ control/auding, solidarity/inclusion/social cohesion, explainability/interpretability, science-policy link, legislative framework/legal status of AI systems, future of employment/worker rights, responsible/intensified research funding, public awareness/education about AI and its risks, dual-use problem/military/AI arms race, field-specific deliberations (health, military, mobility etc.), human autonomy, diversity in the field of AI, cerfication for AI products, protection of whistle blowers, cultural differences in the ethically aligned design of AI systems, and hidden costs (labeling, click-work, contend moderation, energy, resources)
Ryan and Stahl(2021) [19]	ADM	ADM	o	o	o	o	o	o		o	o	o			o		o			Transparency, Justice, Prohibition of Harm (Do No Harm), Responsibility/Accountability, Protection of Privacy, Pursuit of Benefits, Freedom, Trust, Sustainability, Solidarity

Vakkuri et al. (2021) [25]	ADM	ADM	o	o	o	oo	oo	o		o			o	o						Stakeholder Analyze, Transparency, Safe and Security, Fairness, Privacy and Data, Human Agency and Oversight, Wellbeing, Accountability
Mikalef et al.(2022) [22]	ADM	ADM	o	o	oo	o			o		o		o	o	o					Fairness, Transparency, Accountability, Robustness/Safety, Data governance, Laws and regulations, Human oversight, Societal and Environmental wellbeing
Kaur et al. (2022) [21]	ADM	ADM	o	o	oo			o				o								Fairness, Explainability, Accountability, Reliability, Acceptance, Trustworthiness
Stahl et al. (2023) [49]	ADM	ADM	oo			o	oo	ooo		o		o								Unfair and Illegal Discrimination, Privacy; Surveillance Capitalism, Democratic Manipulation, Right to Life, Liberty, and Security, Dignity, AI for Good
Bankins and Formosa(2023) [24]	ADM	ADM	o	o			o	o			o									Beneficence, Non-maleficence, Autonomy, Justice, Explicability
Batool et al.(2025) [50]	ADM	ADM	o	oo		o			o	o			o							Fairness, Privacy and Security, Interpretability, Accuracy, Transparency, Robustness
Gunasekara et al.(2025) [51]	ADM	ADM	oo	oo	o	o		o	o	o		o	o	o						Transparency and Explainability, Fairness and Algorithmic Bias, Privacy and Data Protection, Robustness and Reliability, Accountability, Human Agency and Oversight, Socially Beneficial Practice of AI
Papagiannidis et al.(2025) [52]	ADM	ADM	ooo	o	oo	o	o	o	o	o	o		o	o	o					Accountability, Human Agency and Oversight, Technical Robustness and Safety, Privacy and Data Governance, Transparency, Diversity, Non-discrimination, and Fairness, Societal and Environmental Well-being, Responsible AI

Abbreviation; JF: justice and fairness, T: transparency, R: responsibility, NMS: non-maleficence(safety), W: wellbeing, HR: human right(freedom, autonomy, dignity), TR: robustness, P: privacy, NMI: non-maleficence(prohibition of infringement), CG: common good and trust, DM: data management, C: controllability, SUS: sustainability, I: inclusiveness, S: solidarity, ML: no military abuse, O: openness

Table 3. The referenced priority of AIEPs

Period	Index	JF	T	R	NMS	W	HR	TR	P	NMI	CG	DM	C	SUS	I	S	ML	O	Total
2016-2025	RN	45	39	34	27	26	26	24	23	17	14	13	11	9	6	4	3	2	323
	%	13.93	12.07	10.53	8.36	8.05	8.05	7.43	7.12	5.26	4.33	4.02	3.41	2.79	1.86	1.24	0.93	0.62	100
	ORD	1	2	3	4	5	5	7	8	9	10	11	12	13	14	15	16	17	
2016-2019	RN	26	24	18	16	12	12	16	14	12	9	8	4	3	5	2	1	1	183
	%(①)	14.21	13.11	9.84	8.74	6.56	6.56	8.74	7.65	6.56	4.92	4.37	2.19	1.64	2.73	1.09	0.55	0.55	100
	ORD	1	2	3	4	7	7	4	6	7	10	11	14	13	12	15	16	16	
2020-2025	RN	19	15	16	11	14	14	8	9	5	5	5	7	6	1	2	2	1	140
	%(②)	13.57	10.71	11.43	7.86	10.00	10.00	5.71	6.43	3.57	3.57	3.57	5.00	4.29	0.71	1.43	1.43	0.71	100
	(②-①)	-0.64	-2.40	1.56	-0.89	3.44	3.44	-3.03	-1.22	-2.99	-1.35	-0.80	2.81	2.65	-2.02	0.34	0.88	0.17	
	RN	1	3	2	6	4	4	8	7	11	11	11	9	10	16	15	14	16	
ORD	[17]	2	1	4	3	6	7,10		5	3	6, 8			9		11			
	[18]	2,17,18,21	4,9	3,10,11,13,19	5	12,14,16	6,7,17	5,13	1		6		7,20	6,22		8	15	4	
Matching KAIEP		DV	T	A	NMS	(W)	HR	(TR)	P	NMI	CG	DM	(C)		(I)	S		(O)	

Abbreviation; RN: referenced number, ORD: referenced order, JF: justice and fairness, T: transparency, R: responsibility, NMS: non-maleficence(safety), W: wellbeing, HR: human right(freedom, autonomy, dignity), TR: robustness, P: privacy, NMI: non-maleficence(prohibition of infringement), CG: common good and trust, DM: data management, C: controllability, SUS: sustainability, I: inclusiveness, S: solidarity, ML: no military abuse, O: openness, DV: respect for diversity, A: accountability
* means dropped items in the final version of KAIEPs.

The highest universal priority AIEPs from 2016 to 2025 were JF, T, R, NMS, W, and HR in order of being referenced. Compared to previous studies, the rankings of W and HR have risen slightly, which seems to reflect the changes in people's values after the 2020s [17,18]. These six principles encompass individual, social, and technological values. They also overlap with W, NMS, HR, JF, and T, which are the prerequisites that the All4People framework recommends to reflect them in the actual AI system development [20, 24]. Therefore, we judge these AIEPs as principles that must be reflected in AI system development as much as possible. P, NMI, TR, O, and CG were mentioned very often in previous studies, but in this study, TR was ranked 7th, P was ranked 8th, NMI was ranked 9th, CG was ranked 10th, and O was ranked 17th and was included in the next-lower group. TR stands for the development of artificial intelligence source technology and the provision of stable services. P stands for the right not to be unfairly infringed by others, and NMI stands for the protection of users with the obligation not to infringe on the rights of others. CG means the public interest that artificial intelligence must contribute to the public good. In addition, DM ranked 11th and C ranked 12th are also closely related to data management for system operation, human-centered AI control, and damage prevention, so they are included in the next-lower group. Since the principles included in the next-lower group are the values for the stable operation of artificial intelligence, it is better to respect them as well. Principles belonging to the late group include SUS, I, S, ML, and O. In the case of SUS, the principle is to achieve socioeconomic sustainable growth by minimizing energy consumption and protecting the environment in the process of AI operation. I, S, and O mean social inclusion, and ML means the prohibition of human attacks by AI. There is an opinion that these principles overlap with other principles, so it is better to decide whether to include them or not according to each situation of policy maker.

Efforts have been made to update AIEPs to the current situation. Five years have passed since AIEPs were created by 2020. I looked at what ethical principles have been discussed more actively after 2020 for updating AIEPs. Table 4 shows the difference between the number of mentions of ethical principles before and after 2020 and the percentage of the total mentions. It is interesting to

note that the referenced number of R, W, HR, C, and SUS have increased since 2020. The focus was mainly on creating AIEPs themselves before 2020. Specific guidelines for implementation of ethical principles, legalization, and reflection in actual AI systems were made after 2020. The increase in the referenced number of R explains this difference. Increase in the referenced numbers of HR, W, and C means that there is a growing interest in protecting human basic rights, the need for human-centric AI control, and realizing the benefits of AI to humans in the various areas. Since 2020, interest in AI with humanity has increased. GAI has enabled the general public to feel that they can use AI to increase work productivity. People are now more interested in what AI can do for humans. Since AI is a tool used by humans, human control is needed to reduce unexpected side effects. The increase in the referenced number of SUS emphasized the balance between the use of AI that requires enormous energy and the climate, energy, and environmental protection must be found. AI should contribute to socio-economic sustainable growth. It is also interesting to note that the referenced number of T, TR, NMI, I have decreased since 2020. T and TR are principles that need to be considered continuously in the future because the development of explainable artificial intelligence and the advancement of artificial intelligence technology are necessary. NMI and I are principles for social consideration such as infringement of the rights of others and inclusion of the socially underprivileged. It is regrettable that the mention of these principles has been relatively reduced as previous studies have mainly focused on the development and utilization of artificial intelligence systems since 2020. In summary, I suggest that in order to update AIEPs according to the current situation, the three principles of T, TR, and NMI require continuous to be kept close attention and that the five principles of R, W, HR, C, and SUS should be emphasized for reflecting recently increasing interest.

3.2 Updating KAIEPs and importance-performance analysis

As the use of AI has also spread in Korea, ethical issues such as data bias and misuse of AI have been continuously raised. The Korean government developed AIEPs in 2020 with the goal of promoting 'realizing human-centered AI development'. They defined humanity as the highest value and tried to create AIEPs to achieve three purposes: human dignity, the greater good of society, and the rightful purpose of technology. For three purposes, the Korean government collected the ethical principles mentioned in a total of 25 previous studies and extracted a total of 15 principles such as pursuit of happiness (W), protection of human rights (HR), privacy protection (P), respect for diversity (DV), prohibition of infringement (NMI) for respecting human dignity, such as pursuit of the greater good (CG), openness (O), solidarity (S), inclusiveness (I), data management (DM) for the greater good of society, such as accountability (A), controllability (C), safety (NMS), transparency (T), robustness (TR) for the rightful purpose of technology. Most of the KAIEPs except SUS and ML are included in the 17 integrated AIEPs. Therefore, KAIEPs also have high validity and reliability [33]. Just as the EU used the High-Level Expert Group on Artificial Intelligence (AI HLEG) to create ethical principles for trustworthy AI [13, 52], the Korean government also consulted and collected opinions from the expert group of officers in government, researchers in think tanks, and engineers in bit tech companies. A total of five principles have been deleted since the review of the expert group. If there are too many principles, trade-offs or ethical dilemmas between principles may arise depending on the situation due to duplication and overlap of the content. DV, O, and I are the contents that prohibit unfair discrimination, so they are integrated with DV. C and NMS are integrated into NMS. W is an abstract and comprehensive concept more than other principles, and there is a concern that it may overlap with other principles in that it can be applied to a too wide range of situations. TR has been deleted in response to the expert's point that it is a standard that is difficult to implement in practice at the present time, considering the technical limitations of AI such as uncertainty and unpredictability. Through this process, the final version of 10 KAIEPs of HR, P, DV, NMI, CG, S, DM, R, NMS, and T were confirmed. Table 5 shows the KAIEPs.

Table 4. Description of KAIEPs [33].

Purpose	Principle	Description
Human dignity	W*	AI should contribute to the realization of human happiness by respecting human happiness-seeking tendencies and promoting welfare through quality-of-life improvement.
	HR	AI should respect the basic rights equally granted to all humans. The development and use of AI should not infringe on human rights and freedoms
	P	Anyone should not infringe on the privacy of others or acquire unfair information.
	DV	Bias and discrimination based on individual characteristics such as gender, age, disability, region, race, religion, and country should be minimized. AI should be applied fairly to everyone.
	NMI	AI should not be used for the purpose of causing direct or indirect harm to humans.
Greater good for society	CG	AI should be used not only for the pursuit of personal happiness, but also for the promotion of social publicity and the common benefit of mankind
	O*	Inequality, prejudice, and discrimination based on gender, age, disability, race, religion, and country should be prohibited and everyone should be treated fairly.
	S	It is necessary to maintain relationship solidarity between various groups and utilize AI by fully considering future generations.
	I*	Diversity and inclusion must be maintained in terms of gender, culture, age, professional background and skills
	DM	Data quality and potential risk must be managed throughout data collection and utilization.
Rightful purpose of technology	A	In the process of developing and utilizing artificial intelligence, it is necessary to minimize the damage that can occur by setting up a responsible entity.
	C*	AI must have the ability to immediately control or stop its operation at the judgment of the user during the operation process
	NMS	AI must be built to work safely. When an apparent error or infringement occurs during the operation, the user must be able to control its operation.
	T	It should be able to properly explain the entire execution process of AI.
	TR*	It should be able to operate reliably.

Abbreviation; W: wellbeing, HR: human right, P: privacy, DV: respect for diversity, NMI: prohibition of infringement, CG: common good, O: openness, S: solidarity, I: inclusiveness, DM: data management, A: accountability, C: controllability, NMS: non-maleficence and safety, T: transparency, TR: robustness
* means dropped items in the final version of KAIEPs.

Applying the integrated AIEPs to KAIEPs and updating them is as follows. When referring to the recent changes in the referenced ranking of the integrated AIEPs, it is desirable to revive SUS, W, TR, and C and include them in KAIEPs. SUS was not even included in the original version of KAIEPs, but it has been recently emphasized since 2020. SUS means that AI must be able to contribute to the balance between socio-economic development and environmental protection and to achieve sustainable growth. SUS is a principle that has been treated as important enough to rank 6th out of Hagendorff (2020)'s 22 principles [18]. Korea, in the past, focused on economic development, but recently is interested in ESG management. Therefore, it would be better to newly include SUS in the KAIEPs. W, C, I, and O were excluded from KAIEPs because the concept was too abstract or overlapping with other principles, and TR was excluded from KAIEPs because it was difficult to implement at the current technology level. Reconsideration should be made by reflecting the recent changes such as the general usage on GAI, and W, TR, and C could be revived. W means that the effects of AI should improve various areas of human life. As AI can be used by anyone, such as individuals, companies, and governments, interest in the benefits of AI is increasing significantly. The fact that W's reference ranking has risen to 4th rank and the referenced percentage has also increased by 3.4% since 2020. Article 10 of the Korean Constitution stipulates human dignity, values, and the right to pursue happiness. W should be included in KAIEP. TR is the principle that AI must operate stably even in highly uncertain situations by promoting technological development of AI. TR is considered an important principle to be ranked 7th in the referenced ranking. Technological advancement enables stable service provision of AI, which was not possible in the past. The Korean government is pursuing policy tasks with the aim of becoming one of the top three AI powers. C is integrated with S. C has overlapping meaning with S in terms of being able to immediately minimize damage in an accident, but it also has overlapping meaning with HR in terms of that all activities using AI should be led by humans. C belongs to the lower group at 12th rank in the previous referenced ranking but has recently risen to 7th rank. C is a principle that is emphasized in practice

when developing an AI system and has been frequently referred to since 2020. Since I and O have a low referenced ranking and overlap with DV, the Korean government's judgment that these principles are integrated with DV is reasonable. In summary, I propose that W, TR, and C should be revived, and SUS should be added to the 10 KAIEPs to update them according to the current technological and socio-economic changes. The 14 updated KAIEPs could be DV, T, A, NMS, W, HR, TR, P, NMI, CG, DM, C, SUS, and S.

Around the enforcement of the AI law on January 22, 2026, a tentative evaluation of the adopting performance of 10 KAIEPs was conducted and implications for the future supplementing KAIEPs were drawn. The reason for evaluating 10 KAIEPs instead of the updated 14 principles is that I wanted to measure the actual AI system development performance and compare it with its potential importance. The reason why the previous studies have also simplified the dozens of AIEPs to about five since the 2020s is that it was difficult to reflect too many AIEPs in the actual system development [20]. I conducted an importance-performance analysis to compare the potential importance with the current perceived performance of each KAIEP. Importance-performance analysis is a method often used to derive strategic implications simply by mapping the potential importance and performance of a strategic consideration object to the fourth quadrant [53, 54]. This method has the advantage of being able to clearly understand the strategic direction and resource provision priorities along with the average evaluation of the strategic consideration objects [53]. For the objects located in the first quadrant have higher importance and performance, high performance maintaining strategy should be considered and resources should be allocated in the first order. For the objects located in the second quadrant have high importance but low performance, additional resource allocation and innovation strategies are required to improve current performance. For the objects located in the third quadrant have low importance and performance, and this exit strategy should be considered through business portfolio adjustment. Lastly, the objects located in the fourth quadrant have low importance, but high performance, their importance in the future decreases, so it is necessary to continuously obtain profits but consider gradually reducing the business scope [55]. The importance of KAIEPs was asked "This principle is important, needed, and critical", and the performance was asked on a Likert 7-point scale, "This principle is already well reflected in the AI system, the performance of the AI reflecting KAIEPs is high, and it is well implemented beyond my expectations." on a Likert 7-point scale. A total of 150 practitioners were recruited and required to answer the online survey material. 144 valid responses were obtained. Most of the respondents were employees in their late 20s in their first to fifth years of field operators, majoring in management information system, and had at least one year of AI application development experience. Fig 1 shows the result of the importance-performance analysis for KAIEPs. HR, T, and CG are located in the fourth quadrant, and S is located in the adjacent third quadrant. These principles symbolize the protection of individual basic rights and the value of public interest and democratic solidarity. The theoretical interpretation according to the quadrant position is a strategy to maintain or exit the status quo. However, it should be recalled that all ethical principles have lower performance levels than their importance on average. It is correct to interpret that these principles are well reflected in the system in a balanced way through efforts so far. I hope that efforts to reflect KAIEPs in the development of AI systems will continue in the future to maintain performance so far. In the second quadrant, A, P, NMS, and NMI were located in the order of importance, and DM was located in the third quadrant immediately adjacent to each other. A is a principle of institutionalization and governance for responsible AI. Despite various efforts such as the establishment of ethical guidelines since 2020, the achievements experienced by practitioners are still unclear as these efforts are mainly made through the expert group of AI HLEG. P, NMS, NMI, and DM are the principles for safe AI system development. Despite the importance of these principles, it shows that it is never easy to create a safe AI system that can eliminate or reduce potential risks due to the fundamental non-predictability of AI. In April 2025, when the survey was conducted, private information of about 26.96 million of SK Telecom's subscribers were leaked [56], and in November, private information of about 33.7 million Coupang's customers were leaked [57]. Considering the entire Korean population is 51.6 million in 2025, these events caused a great social impact in Korea. Privacy protection and system security are still considered important issues with

the development of quantum computing and AI technology deployment. In order to improve performance, reflection on the current situation and strategies for innovation and additional resources allocation are needed. In summary, the results of the importance-performance analysis of the 10 KAIEPs show that continuous efforts to supplement the system are needed according to the enforcement of the AI law because overall performance is insufficient compared to the importance in the institutionalization process. It is necessary to continuously reflect the principles of social value that have shown a high level of performance. Efforts to improve performance are urgent for the development of AI governance framework for responsible AI and the adoption of privacy and security principles for safe use of AI.

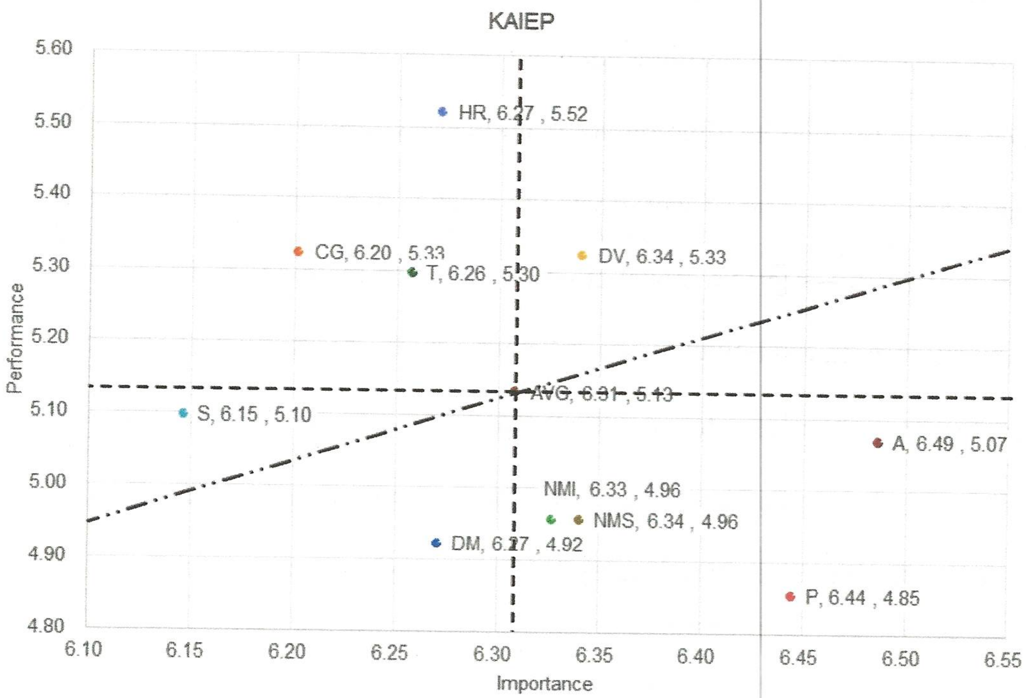

cf. The dotted line represents the importance-performance average of the principles.

Figure 1 importance-performance analysis result.

4. Discussion and Conclusions

By organizing a total of 37 previous studies, I successfully extracted a total of 17 integrated AIEPs: JF, T, R, NMS, W, HR, TR, P, NMI, CG, DM, C, SUS, I, S, ML, and O. Based on the referenced rankings, the universal priorities were determined. In order to maintain timely ethical principles, I proposed constantly to observe whether the three principles of T, TR, and NMI are well applied to the development of AI systems. The five principles of R, W, HR, C, and SUS need to be emphasized and updated. Reflecting the integrated AIEPs, I proposed increasing the existing 10 KAIEPs to 14 by adding W, R, C, and SUS. The results of the importance-performance analysis of KAIEPs suggest that the social value principles of DV, HR, T, CG, and S, which showed high performance levels, should be continuously reflected in the development of AI systems. At the same time, the results show that there is an urgent need to prepare an AI governance system for responsible AI in concerns to A and to develop a privacy protection and security system for safe AI utilization in concerns to P, NMS, NMI, and DM.

ongoing

The academic value of this study is as follows. First, this study extends the research scope, which was mainly focused on AI technology development, to personal and social values and ripple effects that AI should create in the future. With the advancement of AI technology, this is no longer just an automation technology. I am watching numerous AI application services take over even more diverse, complex and detailed human tasks. The more AI is used, the greater the ripple effect that AI has on individuals and society. Along with the question of how AI can be made from the perspective of science and engineering, the question of what AI should be made for from the perspective of humanities and sociology is also important. This is because AI is, after all, a tool for the person who uses it. This study focused on what values AI should create by studying AIEPs for the right purpose of technology, guaranteeing the basic rights of individuals, and promoting the public good of society.

Second, this study balances strategies to increase the positive effects of AI through AI technology development and industrialization, and strategies to minimize side effects by creating regulations for responsible, trustworthy, and safe AI. Many previous studies have been conducted mainly on technology development and user acceptance, focusing on the positive ripple effects of AI. Despite concerns about the fatal threat of AI abuse, only dozens of studies have been conducted to eliminate or minimize side effects. Some people argue that open regulation or non-regulation is more desirable than regulation for the promotion of the AI industry and the spread of services by focusing on the strategic value of AI. This opinion is one of the reasons why the EU's enforcement of AI laws has been delayed. However, from the viewpoint of providing safe services to users and developing the entire industry through fair competition, promotion policies and regulatory policies are not conflicting values. This imbalance must be resolved. This study challenged to reduce the potential risk of AI by conducting research on AIEPs.

Third, this study is one of the very few studies that have proposed integrated AIEPs. Most of the previous studies on AIEPs have created ethical principles in different numbers and contents. It is natural that the ethical principles emphasized are different because history, culture, and ethical values are different for each country, institution, and situation. Since the ethical principles were different for each study, there was no universal framework of AIEPs that countries or companies that needed to create or update AIEPs could refer to. I created 17 integrated AIEPs by combining dozens of existing previous studies. Integrated AIEPs contain all of the most ethical principles mentioned in previous studies. I have made a union and combination of principles that people who want to successfully create AIEPs can refer to.

Fourth, this study is a paper that summarizes the universal priorities between each principle. By compiling the referenced number and rankings of AIEPs, it could be known which ethical principles should be considered first. AIEP with high referenced ranking are principles that must be considered universally, even in various situations. If there is an overlapping conflict between principles, you can refer to ethical principles with high priority. It also helps those who want to create AIEPs to create customized ethical principles, such as different numbers or different content of the ethical principles they want to introduce in each country, organization, and situation.

Fifth, this study created a highly timely ethical principle by updating AIEPs every five years. Most AIEPs were created in 2020. Despite dramatic changes, such as the emergence of ChatGPT in 2022 and the use of AI by the general public, and deployment of AI in industries, these changes have not been reflected in AIEPs. This study calculated and compared the changes in the referenced scores of AIEPs before 2020 and between 2020 and 2025 and found out which principles should be further emphasized. For example, the study argues that R should be emphasized while specific ethical guidelines reflecting ethical principles were created only after 2020 and ensuring consistency between other laws and regulations, that W should be emphasized on how AI can benefit to human as the interest in AI recently increases in the general public, and that SUS should also be emphasized with corresponding to the company's social responsibilities and environment protection in the ESG management practices. Ethical principles must be updated continuously. Therefore, it is hoped that

longitudinal research every five years will continue in subsequent studies in order to maintain timely ethical principles.

Sixth, this study introduced the process of deriving strategic implications of how KAIEPs were made, how they should be updated using integrated AIEPs, and what KAIEPs require more effort to improve progress by comparing the potential importance and tentative performance of each ethical principle. By introducing the world's first case of Korea enforcing AI laws, regulators in other countries who want to implement AI laws later can benchmark them.

The practical value of this study is as follows. First, this study described detailed items of AIEPs in each previous study in [Table 2] for creating integrated AIEPs so that practitioners who want to create or update their own ethical principles can refer to them. By providing specific data, practitioners can contribute to making more sophisticated principles.

Second, this study reorganized KAIEP by referring to the integrated AIEPs. I suggested that the KAIEPs be increased to 14 by adding W, R, C, and SUS. They were dropped at the time of enactment of the KAIEPs in 2020 according to the opinion of AI HLEG, but they have increasing importance to adopt KAIEPs in the actual development of AI systems since 2020. It can lead to various discussions about the roles and limitations of AI HLEG for the improvement of AIEPs. A continuous and constructive discussion to make consensus involving the general public and stakeholders from various fields can help minimize policy failure. Korea's case can also teach other countries a lesson that continuous updates prevent policy failures.

Third, this study conducted an importance-performance analysis of KAIEPs to re-examine ethical principles and derive implications for policy adjustment. Policies must be adjusted through continuous evaluation even after implementation to successfully achieve policy goals and maximize performance. The principles reflecting the social values that have shown a high level of performance should be continuously reflected in the AI system development. It is urgent to prepare a governance system for responsible AI and to reflect the principles of privacy protection and system security for safe AI systems. The arguments of this study support efforts to emphasize the legitimacy of AI regulations and the consistency between laws. At the same time, for practitioners, the need to strengthen privacy protection and system security can explain the necessity of development for these systems that have been marginalized and help secure the legitimacy of budget investment.

Despite these various strengths, this study has several limitations that could not be resolved in research progress. First, this study derived integrated AIEPs using the summary of previous studies. This methodology is not a quantitative research method, and validities of this study could be limited, comparing with other rigorous quantitative methodology such as the laboratory experiment and field survey method. Supplements have been made to enhance the internal validity of methodologies such as using the Preferred Reporting Items for Systematic Reviews and Meta-Analyses (PRISMA) framework. Second, the importance-performance analysis of this study sent an online survey link to each company to have a person in charge of AI system planning and development respond, with 144 respondents, and most of the responses were from practitioners in their mid to late 20s, mainly SMEs. Given the potential importance and investment capacity of AI, subsequent studies will increase the validity of the research results if they receive more responses from companies of various sizes and AI users in various demographic backgrounds. Despite these limitations, this study will be useful as basic data for subsequent research on AIEPs, and it provides many ideas for stakeholders who want to introduce AIEPs. It is hoped that this study will contribute to make the responsible, trustworthy, and safe artificial intelligence systems.

Conflicts of Interest: The author declares no conflict of interest.
