# Supplementary material for: Supplementing Korean artificial intelligence ethical principles: A five-year update
Source: PLoS One. 2026 Aug 21;21(8):e0356766. doi: 10.1371/journal.pone.0356766 (PMC13496415; doi:10.1371/journal.pone.0356766)
Supplement: Supplementary file 4 [file pone.0356766.s005.docx]

AUTHOR RESPONSE (PONE-D-26-06465)

Title: Supplementing Korean Artificial Intelligence Ethical Principles: A Five-year Updates

I would like to thank the editors and reviewers of this paper for their constructive advice to improve the quality of this paper. All reviewers agreed that the paper was timely and necessary for understanding the AI regulation context. I am especially grateful to Reviewer 1 for reading my paper line by line and giving me good advice. Most of the advice went a long way to improving the quality of this paper. I am also grateful to Reviewer 2 for suggesting a new perspective on the value of the paper.I challenged to reflect their advice to revise this paper as it could be possible. I tried to change each line of script; the change had been written in the red character in the revised version. The details of changes were discussed in the table as follows.

| Topic | Reviewer | Comments | Changes |
| --- | --- | --- | --- |
| Editorial Issues | Editor | Please ensure that your manuscript meets PLOS ONE's style requirements, including those for file naming. The PLOS ONE style templates can be found at :  https://journals.plos.org/plosone/s/file?id=wjVg/PLOSOne_formatting_sample_main_body.pdf | Thank you for your comments. I have updated the manuscript title and overall formatting to comply with the PLOS ONE style guidelines. |
|  |  | We note that your Data Availability Statement is currently as follows: “All relevant data are within the manuscript and its Supporting In-formation files.”  Please confirm at this time whether or not your submission contains all raw data required to replicate the results of your study. Authors must share the “minimal data set” for their submission. | For transparency, I have attached the dataset used in our analysis with this revised submission. |
|  |  | Please include a copy of Table 5 which you refer to in your text on page 286. | It was modified to Table 4. |
| Handling acronyms | 1 and 2 | The text, with many acronyms, is hard to follow. The reader will be served if these acronyms are spelled out and greater clarity thereby achieved. | Thank you for your advice. All abbreviations used throughout the manuscript have been expanded to their full forms. For the sake of space and readability, abbreviations have been retained only within the tables. |
|  |  | The manuscript frequently uses abbreviations to refer to each ethical principle. While this may reduce repetition, it can also hinder readability. To improve clarity, the authors may consider providing the full terms alongside the abbreviations—perhaps through foot-notes—so that readers can more easily read this paper. |  |
| Issues in logic development and description | 1 | At line 26, it seems to me that “supplemented” should be replaced with “implemented”.  At line 41, the author cites work predicting that investment in generative AI will reach $644 billion by 2025. I review this document in March 2026, and it would be published only later this year; hence, a reworking of the sentence or an updated figure must be presented to bring this statement up to date.  At lines 53-55, the author writes of “unintended damage to investors” due to the crash of the Robo advisor system. What sort of damage? A bit of detail would be illustrative. Financial losses? Any sense of the magnitude of those losses?  At line 66, the author writes of “apocalypses”. That’s a strong word, suggesting breakdown of society. A weaker term, perhaps “social issues”, might be more appropriate. If the author is directly quoting the CEOs mentioned, then that term should appear in quotation marks to indicate that.  At line 69, what is CAIS?  At line 178, the authors might write that “the implementation of this act, the first policy of its kind, went forward on January 22, 2026. Beyond Korea, AIEPS were…” Similarly at line 92, the implementation of this act is no longer “imminent”, it has been implemented.  In the paragraph starting on line 74, the author makes a number of interesting assertions that would be strengthened with supporting citations at lines 79, 84, and 92.  At line 117 and many places within the manuscript, it is not just the development of AI that is at issue, but also its implementation, which deserves explicit mention.  Part of the potential importance of this review and synthesis is that it would lead readers outside Korea to consider the development of AI oversight policies in their own polity. Hence, citations supporting their access to other policies would be valued. Such citations are needed at lines 126, 128, 129, 131, 133, 138, and 139.  At line 143, what is a “trust foundation”? This may be just a wording issue, which might be addressed by framing it as “the foundation for establishing trust”.  Table 1 is awkwardly labeled. “Taxonomy” is the science of classification. I suggest that the table be labeled Principles of AIEPs.  The author combines methodology, analysis, and results in one section. I find this awkward, as will come out when I write of the importance performance analysis below. The editor might offer advice to the author on whether the journal is OK by combining sections in this manner.  Table 2 might be better labelled as marked on the manuscript. The table is long, spilling over two page breaks, so the reader doesn’t see the definitions for the codes for the AIEPs right way and isn’t told to look for them. Hence, the header might either define the codes up top or tell the reader that they are defined in a footnote. There is no explanation of what the one, two, or three circles in the body of the table represent.  Similarly, Table 4 might be better labeled as marked on the manuscript.  The paragraph starting on line 319 presents the results of importance performance analysis. The reader has not been told of the utility of the method and any software used to perform the analysis. The structure of the output should be explained in general (what it means for a value to be localized in each quadrant) before the reader is led through the results shown in Figure 1. I struggled before I finally was able to follow the text.  A sentence at lines 340-341 is redundant and can be deleted.  A passage now at lines 355-373 would fit better in the Discussion.  At line 357, what is HLEG?  At line 405, the author should add a citation supporting the delay of the EU’s enforcement of AI laws.  At lines 430-435 is a very long sentence that is hard to follow. Can it be broken into perhaps three shorter ones?  At line 471, the author should add a citation supporting the PRISMA framework, which will not be known by most readers. | I sincerely thank you for reviewing my paper line by line. Thank you for all your suggestions. Please note that the line numbers have increased due to the extensive nature of the revisions, which are highlighted in the track-changes version of the manuscript. Based on the advice, lines 33-34 were modified.  Lines 53-54 were modified.  I have added content to clarify that, according to the cited news article, transaction delays prevented customers from responding immediately to market changes at lines 67-69.  Lines 80-81 were modified with the precise phrase "destroy humanity."  I have clarified that CAIS refers to the Center for AI Safety (CAIS, https://aistatement.com/) ). Lines 84 were modified.  Lines 93 and 124-127 were modified to reflect that Korea's AI law had already been implemented.  I have added references that specifically highlight the limitations of previous studies at lines 96-107.  Further explanations have been provided to enhance clarity at lines 134-136.  I have incorporated additional references regarding global government regulatory trends at lines 145, 147, 151, 157, and 160.  The expression has been revised with your suggestion at line 165.  The title has been updated to "Principles of AIEPs” in Table 1.  I agree that separating the methodology from the analysis results significantly improves readability. Accordingly, we have restructured the manuscript: the development of the "Integrated AIEPs" and the IPA analysis are now presented separately under the "Methodology" and "Analysis and Results" sections, respectively.  In the methodology section, I have reinforced the procedural descriptions. Specifically, we added explanatory text at lines 226–231 to define the principles and clarify the meaning of the symbols used in the tables.  I have also verified the title of Table 4.  There is a problem that the readability is poor because there is a description of IPA in the middle of the description. As advised by Reviewer 1, the methodology part was explained in lines 382-401 and a sentence was added to explain the advantages of simplicity of this method.  Regarding lines 340–341, these reflect two distinct survey items measuring "importance" and "performance," respectively.  I believe that the content at lines 355–373 is best placed where it currently resides, as it is essential for interpreting the subsequent IPA analysis. This issue is further addressed in the Discussion section (lines 469–480 and 531–540).  Lines 424-425 were revised.  At lines 476-477, the expression was modified and references were added.  Lines 502-505 were revised to be more concise.  A relevant reference has been added at lines 547. |
| Practical contribution | 2 | This study does not empirically test causal relationships among variables but instead synthesizes prior literature, making it closer to a meta-level or integrative review. Although the PRISMA approach is employed, studies of this type inevitably face limitations in methodological rigor compared to empirical causal research. Therefore, it would be beneficial for the paper to place greater emphasis on its practical contributions rather than theoretical rigor. | I have incorporated a discussion on practical limitations in lines 553-559, as you recommended. |
| Editorial Issues | 2 | Overall, the English in the manuscript communicates the intended meaning clearly. However, some sentences contain overly detailed explanations, slightly awkward phrasing, or stylistic inconsistencies with standard academic writing conventions. A stylistic revision across the manuscript would further improve clarity and readability. | I have thoroughly proofread the entire manuscript to ensure grammatical accuracy and professional academic tone. |
