# Supplementary material for: Supplementing Korean artificial intelligence ethical principles: A five-year update
Source: PLoS One. 2026 Aug 21;21(8):e0356766. doi: 10.1371/journal.pone.0356766 (PMC13496415; doi:10.1371/journal.pone.0356766)
Supplement: Supplementary file 5 [file pone.0356766.s007.docx]

AUTHOR RESPONSE (PONE-D-26-06465)

Title: Supplementing Korean Artificial Intelligence Ethical Principles: A Five-Year Updates

I would like to thank the editors and reviewers of this paper for their constructive advice to improve the quality of this paper. All reviewers agreed that the author has responded well to their comments. Editor and Reviewer 1 requested a revision in several parts to more accurately express the meaning of this paper. The detailed changes are shown in the following table.

| Topic | Reviewer | Comments | Changes |
| --- | --- | --- | --- |
| Editorial Issues | Editor | Line 40: The forecast for 2025 was used as a reference, but since the year has already been in 2026, the reference should be changed to one that presents actual data of 2025. Lines 112–114: Sentences are repeated with analogous meaning. Lines 201–212 and 214–223 : paragraphs are analogous. It would be advisable to merge these two paragraphs into one to eliminate redundant information. Detailed backgrounds and information of the five experts must be provided for readers to fully understand this process. Detailed information regarding three rounds of comprehensive discussion (e.g., timeline, discussion point, etc.) should also be provided. Line 228–253 and 154–282 : they also contains redundant / analogous information. Please Merge these into a single paragraph to create a more cohesive and comprehensive structure. Line 234: Who does “we” refer to? The author? The experts? Lines 385–386: This is not an expression that can be included in the “analysis” section. Please remove it out. Lines 395–365: The survey was stated to have been conducted around the time the law took effect (line 371), but here it is stated to have been conducted in April 2025. This is too significant a discrepancy in timing. Additionally, the case regarding private information leaks mentioned below does not appear to have a direct connection to privacy issues in AI systems. Line 400: Why does quantum computing suddenly come up? Line 464: It cannot be described as “every five years.” So far, it has been a one-time occurrence. | Thank you for your comments. The AI market data and reference have been updated at line 40-41.  It has been omitted at line 116-117.  I strongly agree with your valuable suggestion. Furthermore, Reviewer 1 also raised this point. To fully incorporate both suggestions, I have rewritten and streamlined the 'Methodology, Analysis and Results' sections, which can be found in lines 203–333  (above)  I omitted this sentence.  I omitted this sentence in line 438-439.  I omitted this sentence in line 448-452 and updated references.  I omitted these terms in 453.  I omitted these terms at line 517. |
| Description | 1 | At line 95, the author writes that implementation of the Korean AI law is “imminent”. We are now past the implementation date, so implementation should be characterized as “ongoing”. At line 109, it was recommended BY WHOM to reflect integrated AEIPs on KAIEPs? We need mention of whom and likely also a supporting citation.  Literature review. – Again, at line 149, the Korean AI law is in implementation now. At line 163, we need a numbered citation for Ryan and Stahl (2021). Integrated AIEPs and updates. – At lines 210 and 212, the text refers to Table 2 and then goes straight to mention Table 4 without mentioning Table 3. I’d suggest omitting mention of Table 4 here. There are issues with Table 2 that I identified on the original manuscript 1 that have not been addressed in the revised manuscript. Table 2 might be better understood if presented on pages in landscape orientation, which would then allow the text to be larger. Moreover, the table is long, spilling over two page-breaks, so the reader doesn’t see the definitions for the codes for the AIEPs right way and isn’t told to look for them. Hence, the header might either define the codes up top or tell the reader that they are defined in a footnote. Importantly, there is still no explanation of what the one, two, or three circles in the body of the table represent.  At line 247, should “late” be “last”?  Updating KAIEPs. – At line 320, what is “ESG”? Did I miss it, or is this acronym not defined?  Importance-performance analysis. – At lines 383-384, the authors write that “all ethical principles have lower performance levels than their importance”. On what is this important assertion based? The author should support the statement by citing his own data or with a supporting citation from the literature.  Discussion and Conclusions. – At line 501, would “systems” be better as “considerations”? | I sincerely thank you for reviewing my paper line by line. Thank you for all your suggestions. The term has been changed to ” now imminent in EU and ongoing in Korea,” at line 96.  This part is a description in which the author suggested ideas to policy agencies, so there is no reference. Therefore, the tone of the sentence was adjusted to suit the meaning at lines 109-111.  The term has been changed to ”has been” in line 151.  Numbered citation has been added at line 166.  I omitted mention of Table 4 at line 213-214.  In response to Reviewer 1's comment, I previously added lines 217–218 in Revision 1 ('Experts...Table 4.'). Furthermore, in that added sentence (lines 211–212), I already explained that the number of circles in the table body represents the count of codes included in each ethical principle. Nevertheless, the repetition of this same point suggests that our description in the methodology section was still insufficient. As the Editor has also raised a similar concern, I have substantially revised and elaborated on the methodology section in lines 203-333. Moreover, the suggestion to present Table 2 in landscape orientation to improve readability is well taken. I will move the 'codes' of the final column in Table 2 to a footnote, present Table 2 in a landscape format, and add an explanation in the main text directing readers for the definitions of the codes included in each principle. However, because the footnote content was too long and caused the table to break across pages, the footnote was placed immediately beneath the table to enhance readability.  The term has been changed to ”last” at line 247.  The term has been changed to ”business practices of environmental, social, and governance consideration” at line 371.  The sentence has been changed to ” However, it should be noted that the importance ratings for all ten ethical principles consistently exceed their performance ratings, and the overall mean of importance is higher than that of performance.” at 434-436.  The term ”systems” has been changed to "considerations” at line 554 to avoid redundancy. |
