# Supplementary material for: Supplementing Korean artificial intelligence ethical principles: A five-year update
Source: PLoS One. 2026 Aug 21;21(8):e0356766. doi: 10.1371/journal.pone.0356766 (PMC13496415; doi:10.1371/journal.pone.0356766)
Supplement: Peer review history

# Peer review: Decision Letter 0

**DOI:** 10.1371/journal.pone.0356766.r001

**Authors:** Hongbum Kim

4 Apr 2026

Dear Dr. Lee,

Thank you for submitting your manuscript to PLOS ONE. After careful consideration, we feel that it has merit but does not fully meet PLOS ONE’s publication criteria as it currently stands. Therefore, we invite you to submit a revised version of the manuscript that addresses the points raised during the review process.

Please submit your revised manuscript by May 19 2026 11:59PM. If you will need more time than this to complete your revisions, please reply to this message or contact the journal office at plosone@plos.org. When you're ready to submit your revision, log on to https://www.editorialmanager.com/pone/ and select the 'Submissions Needing Revision' folder to locate your manuscript file.

- A letter that responds to each point raised by the academic editor and reviewer(s). You should upload this letter as a separate file labeled 'Response to Reviewers'.
- A marked-up copy of your manuscript that highlights changes made to the original version. You should upload this as a separate file labeled 'Revised Manuscript with Track Changes'.
- An unmarked version of your revised paper without tracked changes. You should upload this as a separate file labeled 'Manuscript'.

If you would like to make changes to your financial disclosure, please include your updated statement in your cover letter. Guidelines for resubmitting your figure files are available below the reviewer comments at the end of this letter.

If applicable, we recommend that you deposit your laboratory protocols in protocols.io to enhance the reproducibility of your results. Protocols.io assigns your protocol its own identifier (DOI) so that it can be cited independently in the future. For instructions see: https://journals.plos.org/plosone/s/submission-guidelines#loc-laboratory-protocols. Additionally, PLOS ONE offers an option for publishing peer-reviewed Lab Protocol articles, which describe protocols hosted on protocols.io. Read more information on sharing protocols at https://plos.org/protocols?utm_medium=editorial-email&utm_source=authorletters&utm_campaign=protocols.

As the corresponding author, your ORCID iD is verified in the submission system and will appear in the published article. PLOS supports the use of ORCID, and we encourage all coauthors to register for an ORCID iD and use it as well. Please encourage your coauthors to verify their ORCID iD within the submission system before final acceptance, as unverified ORCID iDs will not appear in the published article. *Only*  the individual author can complete the verification step; PLOS staff *cannot*  verify ORCID iDs on behalf of authors.

We look forward to receiving your revised manuscript.

Kind regards,

Hongbum Kim, Ph.D.

Academic Editor

PLOS One

Journal Requirements:

When submitting your revision, we need you to address these additional requirements.

1.Please ensure that your manuscript meets PLOS ONE's style requirements, including those for file naming. The PLOS ONE style templates can be found at

https://journals.plos.org/plosone/s/file?id=wjVg/PLOSOne_formatting_sample_main_body.pdf and

https://journals.plos.org/plosone/s/file?id=ba62/PLOSOne_formatting_sample_title_authors_affiliations.pdf

2. We note that your Data Availability Statement is currently as follows: “All relevant data are within the manuscript and its Supporting Information files.”

Please confirm at this time whether or not your submission contains all raw data required to replicate the results of your study. Authors must share the “minimal data set” for their submission. PLOS defines the minimal data set to consist of the data required to replicate all study findings reported in the article, as well as related metadata and methods (https://journals.plos.org/plosone/s/data-availability#loc-minimal-data-set-definition).

For example, authors should submit the following data:

- The values behind the means, standard deviations and other measures reported;

- The values used to build graphs;

- The points extracted from images for analysis.

Authors do not need to submit their entire data set if only a portion of the data was used in the reported study.

If your submission does not contain these data, please either upload them as Supporting Information files or deposit them to a stable, public repository and provide us with the relevant URLs, DOIs, or accession numbers. For a list of recommended repositories, please see https://journals.plos.org/plosone/s/recommended-repositories.

If there are ethical or legal restrictions on sharing a de-identified data set, please explain them in detail (e.g., data contain potentially sensitive information, data are owned by a third-party organization, etc.) and who has imposed them (e.g., an ethics committee). Please also provide contact information for a data access committee, ethics committee, or other institutional body to which data requests may be sent. If data are owned by a third party, please indicate how others may request data access.

3. Please include a copy of Table 5 which you refer to in your text on page 286.

4. If the reviewer comments include a recommendation to cite specific previously published works, please review and evaluate these publications to determine whether they are relevant and should be cited. There is no requirement to cite these works unless the editor has indicated otherwise.

Additional Editor Comments:

Please check the attached file provided by Reviewer for your revision as well.

[Note: HTML markup is below. Please do not edit.]

Reviewer's Responses to Questions

**Comments to the Author**

1. Is the manuscript technically sound, and do the data support the conclusions?

Reviewer #1: Yes

Reviewer #2: Yes

**********

2. Has the statistical analysis been performed appropriately and rigorously?

Reviewer #1: Yes

Reviewer #2: Yes

**********

3. Have the authors made all data underlying the findings in their manuscript fully available?

The PLOS Data policy

Reviewer #1: No

Reviewer #2: Yes

**********

4. Is the manuscript presented in an intelligible fashion and written in standard English?

Reviewer #1: No

Reviewer #2: Yes

**********

Reviewer #1: Artificial intelligence is being developed and implemented in a range of applications with surprising speed. People are rightfully concerned about possible negative impacts, and governments have been slow to respond with regulation. Even the ethical principles upon which regulatory oversight would be based are not agreed upon. Lee reviews the development of AI ethical principles, synthesizing Korean and international developments. The insights are potentially important beyond Korea, justifying publication of this review and synthesis in an international journal. The manuscript before me will need clean-up and sharpening to realize its full potential impact.

- The text, with many acronyms, is hard to follow. The reader will be served if these acronyms are spelled out and greater clarity thereby achieved.

- The prose is rough. I appreciate that the author is writing in a second language. I have marked the text to help the author develop sharper prose. I hope the marks are taken as constructive.

- Some passages will need to be updated, as detailed below.

- I suggest that methods be presented in a stand-alone Methods section.

Context-dependent comments follow.

Abstract. – At line 26, it seems to me that “supplemented” should be replaced with “implemented”.

Introduction. – At line 41, the author cites work predicting that investment in generative AI will reach $644 billion by 2025. I review this document in March 2026, and it would be published only later this year; hence, a reworking of the sentence or an updated figure must be presented to bring this statement up to date.

At lines 53-55, the author writes of “unintended damage to investors” due to the crash of the Robo advisor system. What sort of damage? A bit of detail would be illustrative. Financial losses? Any sense of the magnitude of those losses?

At line 66, the author writes of “apocalypses”. That’s a strong word, suggesting breakdown of society. A weaker term, perhaps “social issues”, might be more appropriate. If the author is directly quoting the CEOs mentioned, then that term should appear in quotation marks to indicate that.

At line 69, what is CAIS?

Again, events have overtaken the submission of this manuscript. Hence, at line 178, the authors might write that “the implementation of this act, the first policy of its kind, went forward on January 22, 2026. Beyond Korea, AIEPS were…” Similarly at line 92, the implementation of this act is no longer “imminent”, it has been implemented.

In the paragraph starting on line 74, the author makes a number of interesting assertions that would be strengthened with supporting citations at lines 79, 84, and 92.

Literature review. - At line 117 and many places within the manuscript, it is not just the development of AI that is at issue, but also its implementation, which deserves explicit mention.

Part of the potential importance of this review and synthesis is that it would lead readers outside Korea to consider the development of AI oversight policies in their own polity. Hence, citations supporting their access to other policies would be valued. Such citations are needed at lines 126, 128, 129, 131, 133, 138, and 139.

At line 143, what is a “trust foundation”? This may be just a wording issue, which might be addressed by framing it as “the foundation for establishing trust”.

Table 1 is awkwardly labeled. “Taxonomy” is the science of classification. I suggest that the table be labeled Principles of AIEPs.

Methods and Results. – The author combines methodology, analysis, and results in one section. I find this awkward, as will come out when I write of the importance performance analysis below. The editor might offer advice to the author on whether the journal is OK by combining sections in this manner.

Table 2 might be better labelled as marked on the manuscript. The table is long, spilling over two page breaks, so the reader doesn’t see the definitions for the codes for the AIEPs right way and isn’t told to look for them. Hence, the header might either define the codes up top or tell the reader that they are defined in a footnote. There is no explanation of what the one, two, or three circles in the body of the table represent.

Similarly, Table 4 might be better labeled as marked on the manuscript.

The paragraph starting on line 319 presents the results of importance performance analysis. The reader has not been told of the utility of the method and any software used to perform the analysis. The structure of the output should be explained in general (what it means for a value to be localized in each quadrant) before the reader is led through the results shown in Figure 1. I struggled before I finally was able to follow the text.

A sentence at lines 340-341 is redundant and can be deleted.

A passage now at lines 355-373 would fit better in the Discussion.

At line 357, what is HLEG?

Discussion and Conclusions. – At line 405, the author should add a citation supporting the delay of the EU’s enforcement of AI laws.

At lines 430-435 is a very long sentence that is hard to follow. Can it be broken into perhaps three shorter ones?

At line 471, the author should add a citation supporting the PRISMA framework, which will not be known by most readers.

Reviewer #2: Overall Evaluation

This study successfully identifies 17 elements of artificial intelligence (AI) ethical principles by synthesizing prior academic research and conducting cross-national comparisons. By considering both the frequency and priority with which these principles are discussed in the literature, the study attempts to derive universal ethical principles that transcend national and cultural differences. The results are subsequently applied to the Korean AI ethical principles to propose directions for its future revision. As such, the study provides a valuable foundation for future research and offers meaningful reference material for scholars and policymakers. Moreover, given that Korea plans to implement both an AI Act and AI ethical principles in 2026—the first such case globally—the study is both timely and relevant. Nevertheless, addressing the following three points would further strengthen the persuasiveness of the findings.

1. Clarification of the Study’s Practical Contribution

This study does not empirically test causal relationships among variables but instead synthesizes prior literature, making it closer to a meta-level or integrative review. Although the PRISMA approach is employed, studies of this type inevitably face limitations in methodological rigor compared to empirical causal research. Therefore, it would be beneficial for the paper to place greater emphasis on its practical contributions rather than theoretical rigor.

In the introduction, the authors state that the study aims to address several limitations of existing AI ethical principles. In addition to the four limitations discussed in the third paragraph of the introduction, another important issue could be emphasized: although there are only about ten major AI ethical principles, the number of detailed practical guidelines derived from them approaches one hundred, and the number of operational directives can be even larger. Because these principles increasingly carry quasi-regulatory force and influence corporate compliance, organizations must respond quickly. Given that companies often have only a few months to adapt to AI regulations and ethical principles, the need to update and selectively adopt existing AI ethical guidelines is both urgent and practically significant. Highlighting this point more clearly in the introduction, discussion, and conclusion would strengthen the study’s practical relevance.

2. Use of Abbreviations

The manuscript frequently uses abbreviations to refer to each ethical principle. While this may reduce repetition, it can also hinder readability. To improve clarity, the authors may consider providing the full terms alongside the abbreviations—perhaps through footnotes—so that readers can more easily read this paper.

3. Language and Editorial Refinement

Overall, the English in the manuscript communicates the intended meaning clearly. However, some sentences contain overly detailed explanations, slightly awkward phrasing, or stylistic inconsistencies with standard academic writing conventions. A stylistic revision across the manuscript would further improve clarity and readability.

Recommendation

Given that this paper addresses the very early stage of AI regulatory implementation and provides a timely synthesis of AI ethical principles, it is likely to serve as a useful reference for subsequent research and policy discussions. For this reason, I recommend expediting its publication after minor revisions.

**********

what does this mean?). If published, this will include your full peer review and any attached files.

If you choose “no”, your identity will remain anonymous but your review may still be made public.

**Do you want your identity to be public for this peer review?**  For information about this choice, including consent withdrawal, please see our Privacy Policy

Reviewer #1: No

Reviewer #2: No

**********

[NOTE: If reviewer comments were submitted as an attachment file, they will be attached to this email and accessible via the submission site. Please log into your account, locate the manuscript record, and check for the action link "View Attachments". If this link does not appear, there are no attachment files.]

To ensure your figures meet our technical requirements, please review our figure guidelines: https://journals.plos.org/plosone/s/figures

You may also use PLOS’s free figure tool, NAAS, to help you prepare publication quality figures: https://journals.plos.org/plosone/s/figures#loc-tools-for-figure-preparation.

NAAS will assess whether your figures meet our technical requirements by comparing each figure against our figure specifications.

Attachment Submitted filename: Lee.pdf

# Author comment: Author response to Decision Letter 1

**DOI:** 10.1371/journal.pone.0356766.r002

31 May 2026

I would like to thank the editors and reviewers of this paper for their constructive advice to improve the quality of this paper. All reviewers agreed that the paper was timely and necessary for understanding the AI regulation context. I am especially grateful to Reviewer 1 for reading my paper line by line and giving me good advice. Most of the advice went a long way to improving the quality of this paper. I am also grateful to Reviewer 2 for suggesting a new perspective on the value of the paper.I challenged to reflect their advice to revise this paper as it could be possible. I tried to change each line of script; the change had been written in the red character in the revised version. The details of changes were discussed in the attached file named Response to Reviewers PONE-D-26-06465_R1.

Attachment Submitted filename: Response to Reviewers PONE-D-26-06465_R1.docx

# Peer review: Decision Letter 1

**DOI:** 10.1371/journal.pone.0356766.r003

**Authors:** Hongbum Kim

14 Jun 2026

Dear Dr. Lee,

Thank you for submitting your manuscript to PLOS ONE. After careful consideration, we feel that it has merit but does not fully meet PLOS ONE’s publication criteria as it currently stands. Therefore, we invite you to submit a revised version of the manuscript that addresses the points raised during the review process.

Please submit your revised manuscript by Jul 29 2026 11:59PM. If you will need more time than this to complete your revisions, please reply to this message or contact the journal office at plosone@plos.org. When you're ready to submit your revision, log on to https://www.editorialmanager.com/pone/ and select the 'Submissions Needing Revision' folder to locate your manuscript file.

- A letter that responds to each point raised by the academic editor and reviewer(s). You should upload this letter as a separate file labeled 'Response to Reviewers'.
- A marked-up copy of your manuscript that highlights changes made to the original version. You should upload this as a separate file labeled 'Revised Manuscript with Track Changes'.
- An unmarked version of your revised paper without tracked changes. You should upload this as a separate file labeled 'Manuscript'.

If you would like to make changes to your financial disclosure, please include your updated statement in your cover letter. Guidelines for resubmitting your figure files are available below the reviewer comments at the end of this letter.

If applicable, we recommend that you deposit your laboratory protocols in protocols.io to enhance the reproducibility of your results. Protocols.io assigns your protocol its own identifier (DOI) so that it can be cited independently in the future. For instructions see: https://journals.plos.org/plosone/s/submission-guidelines#loc-laboratory-protocols. Additionally, PLOS ONE offers an option for publishing peer-reviewed Lab Protocol articles, which describe protocols hosted on protocols.io. Read more information on sharing protocols at https://plos.org/protocols?utm_medium=editorial-email&utm_source=authorletters&utm_campaign=protocols.

As the corresponding author, your ORCID iD is verified in the submission system and will appear in the published article. PLOS supports the use of ORCID, and we encourage all coauthors to register for an ORCID iD and use it as well. Please encourage your coauthors to verify their ORCID iD within the submission system before final acceptance, as unverified ORCID iDs will not appear in the published article. *Only*  the individual author can complete the verification step; PLOS staff *cannot*  verify ORCID iDs on behalf of authors.

We look forward to receiving your revised manuscript.

Kind regards,

Hongbum Kim, Ph.D.

Academic Editor

PLOS One

**Journal Requirements:**

1. If the reviewer comments include a recommendation to cite specific previously published works, please review and evaluate these publications to determine whether they are relevant and should be cited. There is no requirement to cite these works unless the editor has indicated otherwise.

2. Please review your reference list to ensure that it is complete and correct. If you have cited papers that have been retracted, please include the rationale for doing so in the manuscript text, or remove these references and replace them with relevant current references. Any changes to the reference list should be mentioned in the rebuttal letter that accompanies your revised manuscript. If you need to cite a retracted article, indicate the article’s retracted status in the References list and also include a citation and full reference for the retraction notice.

**Additional Editor Comments:**

Including additional comments from Reviewer #1 and the detailed corrections in the attached files, the following suggestions should be considered for the publication.

Line 40: The forecast for 2025 was used as a reference, but since the year has already been in 2026, the reference should be changed to one that presents actual data of 2025.

Lines 112–114: Sentences are repeated with analogous meaning.

Lines 201–212 and 214–223 : paragraphs are analogous. It would be advisable to merge these two paragraphs into one to eliminate redundant information. Detailed backgrounds and information of the five experts must be provided for readers to fully understand this process. Detailed information regarding three rounds of comprehensive discussion (e.g., timeline, discussion point, etc.) should also be provided.

Line 228–253 and 154–282 : they also contains redundant / analogous information. Please Merge these into a single paragraph to create a more cohesive and comprehensive structure.

Line 234: Who does “we” refer to? The author? The experts?

Lines 385–386: This is not an expression that can be included in the “analysis” section. Please remove it out.

Lines 395–365: The survey was stated to have been conducted around the time the law took effect (line 371), but here it is stated to have been conducted in April 2025. This is too significant a discrepancy in timing. Additionally, the case regarding private information leaks mentioned below does not appear to have a direct connection to privacy issues in AI systems.

Line 400: Why does quantum computing suddenly come up?

Line 464: It cannot be described as “every five years.” So far, it has been a one-time occurrence.

[Note: HTML markup is below. Please do not edit.]

Reviewers' comments:

Reviewer's Responses to Questions

**Comments to the Author**

Reviewer #1: (No Response)

Reviewer #2: All comments have been addressed

**********

2. Is the manuscript technically sound, and do the data support the conclusions?

Reviewer #1: Yes

Reviewer #2: Yes

**********

3. Has the statistical analysis been performed appropriately and rigorously?

Reviewer #1: Yes

Reviewer #2: N/A

**********

4. Have the authors made all data underlying the findings in their manuscript fully available?

The PLOS Data policy

Reviewer #1: No

Reviewer #2: Yes

**********

5. Is the manuscript presented in an intelligible fashion and written in standard English?

Reviewer #1: No

Reviewer #2: Yes

**********

**Reviewer #1:**  Artificial intelligence is being developed and implemented in a range of applications with surprising speed. People are rightfully concerned about possible negative impacts, and governments have been slow to respond with regulation. Even the ethical principles upon which regulatory oversight would be based are not agreed upon. Lee reviews the development of AI ethical principles, synthesizing Korean and international developments. The insights are potentially important beyond Korea, justifying publication of this review and synthesis in an international journal. The manuscript before me is a revised one, and is notably strengthened. While improved, the prose will need further sharpening to improve readability and realize its full potential impact. I have once again marked the text to help the author develop sharper prose. I hope the marks are taken as constructive as the author prepares a second and hopefully final revision.

Context-dependent comments follow.

Introduction. – At line 95, the author writes that implementation of the Korean AI law is “imminent”. We are now past the implementation date, so implementation should be characterized as “ongoing”.

At line 109, it was recommended BY WHOM to reflect integrated AEIPs on KAIEPs? We need mention of whom and likely also a supporting citation.

Literature review. – Again, at line 149, the Korean AI law is in implementation now.

At line 163, we need a numbered citation for Ryan and Stahl (2021).

Integrated AIEPs and updates. – At lines 210 and 212, the text refers to Table 2 and then goes straight to mention Table 4 without mentioning Table 3. I’d suggest omitting mention of Table 4 here.

There are issues with Table 2 that I identified on the original manuscript 1 that have not been addressed in the revised manuscript. Table 2 might be better understood if presented on pages in landscape orientation, which would then allow the text to be larger. Moreover, the table is long, spilling over two page-breaks, so the reader doesn’t see the definitions for the codes for the AIEPs right way and isn’t told to look for them. Hence, the header might either define the codes up top or tell the reader that they are defined in a footnote. Importantly, there is still no explanation of what the one, two, or three circles in the body of the table represent.

At line 247, should “late” be “last”?

Updating KAIEPs. – At line 320, what is “ESG”? Did I miss it, or is this acronym not defined?

Importance-performance analysis. – At lines 383-384, the authors write that “all ethical principles have lower performance levels than their importance”. On what is this important assertion based? The author should support the statement by citing his own data or with a supporting citation from the literature.

Discussion and Conclusions. – At line 501, would “systems” be better as “considerations”?

**Reviewer #2:**  The authors have well responded to my comments. Overall, the author have made an enough effort to improve the manuscript.

**********

what does this mean?). If published, this will include your full peer review and any attached files.

If you choose “no”, your identity will remain anonymous but your review may still be made public.

**Do you want your identity to be public for this peer review?**  For information about this choice, including consent withdrawal, please see our Privacy Policy

Reviewer #1: No

Reviewer #2: No

**********

[NOTE: If reviewer comments were submitted as an attachment file, they will be attached to this email and accessible via the submission site. Please log into your account, locate the manuscript record, and check for the action link "View Attachments". If this link does not appear, there are no attachment files.]

To ensure your figures meet our technical requirements, please review our figure guidelines: https://journals.plos.org/plosone/s/figures

You may also use PLOS’s free figure tool, NAAS, to help you prepare publication quality figures: https://journals.plos.org/plosone/s/figures#loc-tools-for-figure-preparation.

NAAS will assess whether your figures meet our technical requirements by comparing each figure against our figure specifications.

Attachment Submitted filename: Lee.pdf

# Author comment: Author response to Decision Letter 2

**DOI:** 10.1371/journal.pone.0356766.r004

27 Jul 2026

I would like to thank the editors and reviewers of this paper for their constructive advice to improve the quality of this paper. All reviewers agreed that the author has responded well to their comments. Editor and Reviewer 1 requested a revision in several parts for carring more accurate meaning of this paper. The detailed changes are shown in the table at the response to reviewers of the attached file.

Attachment Submitted filename: Response to Reviewers PONE-D-26-06465_R2.docx

# Peer review: Decision Letter 2

**DOI:** 10.1371/journal.pone.0356766.r005

**Authors:** Hongbum Kim

10 Aug 2026

Supplementing Korean Artificial Intelligence Ethical Principles: A Five-year Updates

PONE-D-26-06465R2

Dear Dr. Lee,

We’re pleased to inform you that your manuscript has been judged scientifically suitable for publication and will be formally accepted for publication once it meets all outstanding technical requirements.

Within one week, you’ll receive an e-mail detailing the required amendments. When these have been addressed, you’ll receive a formal acceptance letter and your manuscript will be scheduled for publication.

An invoice will be generated when your article is formally accepted. Please note, if your institution has a publishing partnership with PLOS and your article meets the relevant criteria, all or part of your publication costs will be covered. Please make sure your user information is up-to-date by logging into Editorial Manager at Editorial Manager® and clicking the ‘Update My Information' link at the top of the page. For questions related to billing, please contact billing support.

If your institution or institutions have a press office, please notify them about your upcoming paper to help maximize its impact. If they’ll be preparing press materials, please inform our press team as soon as possible -- no later than 48 hours after receiving the formal acceptance. Your manuscript will remain under strict press embargo until 2 pm Eastern Time on the date of publication. For more information, please contact onepress@plos.org.

Kind regards,

Hongbum Kim, Ph.D.

Academic Editor

PLOS One

Additional Editor Comments (optional):

I am sincerely thankful for your hard work in enhancing the quality of your manuscript. I have decided to accept your paper, but I ask you to incorporate the additional comments from Reviewer #2, including  an attached file, during the following editing process. Congratulations.

Reviewers' comments:

Reviewer's Responses to Questions

**Comments to the Author**

Reviewer #1: (No Response)

**********

2. Is the manuscript technically sound, and do the data support the conclusions?

Reviewer #1: Yes

**********

3. Has the statistical analysis been performed appropriately and rigorously?

Reviewer #1: Yes

**********

4. Have the authors made all data underlying the findings in their manuscript fully available?

The PLOS Data policy

Reviewer #1: Yes

**********

5. Is the manuscript presented in an intelligible fashion and written in standard English?

Reviewer #1: No

**********

Reviewer #1: Artificial intelligence is being developed and implemented in a range of applications with surprising speed. People are rightfully concerned about possible negative impacts, and governments have been slow to respond with regulation. Even the ethical principles upon which regulatory oversight would be based are not agreed upon. Lee reviews the development of AI ethical principles, synthesizing Korean and international developments. The insights are potentially important beyond Korea, justifying publication of this review and synthesis in an international journal. The manuscript before me is a twice-revised one, and is just about ready for acceptance. The prose will need final sharpening to improve readability and realize its full potential impact. I have once again marked the text to help the author develop the final prose revisions. One issue came up repeatedly: “human rights” should always be plural.

Context-dependent comments follow.

Title: “update” should be singular.

Introduction. – The acronym GAI must be defined at first usage at line 41.

Integrated AIEPs and updates. – Table 3 appears in the wrong place. It should follow its first mention and appear at line 255.

At line 239, harm can be prevented, but risk cannot. Risk is the likelihood that harm will be realized given that an AI user is exposed to the hazard, AI. Hence, risk cannot be “prevented”, it must be “minimized”.

Updating KAIEPs. – At line 301, insertion of an adjective – perhaps “current”, “revised”, or “updated” – would add clarity to the sentence.

Importance-performance analysis. – The description of the four quadrants in the depiction of the results of importance-performance analysis would benefit from a small modification that would increase clarity. For example, at line 346, which is the “first” quadrant, the upper left one? At line 348, which is the “second” quadrant, etc., for all four quadrants? The reader should not be left to make the inference. The text can be changed and perhaps also the respective quadrants in Figure 1 can be labeled.

Discussion and Conclusions. – the acronym SMEs at line 497 must be defined.

**********

what does this mean?). If published, this will include your full peer review and any attached files.

If you choose “no”, your identity will remain anonymous but your review may still be made public.

**Do you want your identity to be public for this peer review?**  For information about this choice, including consent withdrawal, please see our Privacy Policy

Reviewer #1: No

**********

Attachment Submitted filename: Lee.pdf

# Editor report: Acceptance letter

**DOI:** 10.1371/journal.pone.0356766.r006

**Authors:** Hongbum Kim

PONE-D-26-06465R2

PLOS One

Dear Dr. Lee,

I'm pleased to inform you that your manuscript has been deemed suitable for publication in PLOS One. Congratulations! Your manuscript is now being handed over to our production team.

At this stage, our production department will prepare your paper for publication. This includes ensuring the following:

* All references, tables, and figures are properly cited

* All relevant supporting information is included in the manuscript submission,

* There are no issues that prevent the paper from being properly typeset

You will receive further instructions from the production team, including instructions on how to review your proof when it is ready. Please keep in mind that we are working through a large volume of accepted articles, so please give us a few days to review your paper and let you know the next and final steps.

Lastly, if your institution or institutions have a press office, please let them know about your upcoming paper now to help maximize its impact. If they'll be preparing press materials, please inform our press team within the next 48 hours. Your manuscript will remain under strict press embargo until 2 pm Eastern Time on the date of publication. For more information, please contact onepress@plos.org.

You will receive an invoice from PLOS for your publication fee after your manuscript has reached the completed accept phase. If you receive an email requesting payment before acceptance or for any other service, this may be a phishing scheme. Learn how to identify phishing emails and protect your accounts at https://explore.plos.org/phishing.

If we can help with anything else, please email us at customercare@plos.org.

Thank you for submitting your work to PLOS One and supporting open access.

Kind regards,

PLOS ONE Editorial Office Staff

on behalf of

Professor Hongbum Kim

Academic Editor

PLOS One
